# Improving Preservice Primary Teachers’ Understanding of the Nature of Methods of Science Through Reflective Reading of News Articles

**DOI:** 10.1007/s11191-022-00338-y

**Published:** 2022-05-13

**Authors:** Antonio García-Carmona

**Affiliations:** Departamento de Didáctica de las Ciencias Experimentales y Sociales, Universidad de Sevilla, Seville, Spain

**Keywords:** Media, Methods of science, Nature of science, Reading, Teacher education, The scientific method

## Abstract

A study is presented that analyzed the pedagogical efficacy of reading opinion articles about methods of science, published in the media, in order to improve the meta-scientific understanding of 52 preservice primary teachers (PPTs) with regard to the topic. To this end, an activity was designed taking an explicit and reflective approach. The design of the activity required a short teaching intervention when being implemented in class in order to facilitate its integration into the program of the subject of science teaching. Before doing the activity, the PPTs’ prior conceptions about the nature of methods of science were diagnosed using the *Opinions about Science, Technology and Society Questionnaire* (COCTS, in its Spanish acronym). The activity consisted of reading the articles, and then responding in small work groups to a series of questions for reflection and debate on the topic. The groups’ responses were then shared and discussed in class. Once the activity had finished, the PPTs responded to the questionnaire again (post-test) in order to evaluate how their conceptions had progressed. There was an improvement in their understanding of various aspects of the nature of methods of science (e.g., scientists use a variety of methods in their research depending on the object of study, the context, and the resources available, or that *the scientific method* is an idealized, simplistic, and therefore poor representation of how scientists do research). These results show that the activity was effective in getting the PPTs to reflect and learn about the topic. Finally, the limitations of the study are discussed (e.g., the limited time frame to implement the activity and evaluate results), and some future research perspectives are given to improve the understanding of PPTs about the nature of methods of science.

## Introduction

Any citizen science literacy plan should include among its main objectives the attainment of a basic understanding of what science is, how it works, what its limits are, and what factors influence its development as a human activity (Bybee et al., 1991; Hodson, 2009; NSTA 2020). This polyhedral *metaknowledge*, known as the *nature of science* (NOS), derives from studies about the history, philosophy, and sociology of science, as well as some contributions from the cognitive sciences (McComas & Clough, 2020). The delimitation and adaptation of NOS content to the educational context is therefore a pedagogically complex issue, which is a matter of permanent discussion in science education (Hodson & Wong, 2014; Kampourakis, 2016; Wallace, 2017). Even so, one can find interesting and viable proposals in the international literature to introduce NOS content into the school science curriculum (e.g., Erduran & Dagher, 2014; García-Carmona & Acevedo-Díaz, 2018; Lederman, 2007).

Multiple reasons can be given to justify attention to NOS in science education (Driver et al., 1996; Science Learning Hub, 2011), but one of especial relevance is the need to prepare citizens to be able to participate critically and responsibly in debates and decision-making about matters related to science. It is true that, when analyzing these issues, scientific and meta-scientific arguments tend to overlap with those of another nature, such as political, economic, psychological, emotional, or ethical/moral (Karisan & Zeidler, 2017). Nonetheless, it has been found that when there is a lack of informed understanding of NOS, non-scientific arguments often prevail over scientific arguments in such debates (Bell & Lederman, 2003). This is really worrying because it encourages science deniers and pseudoscience promoters to acquire social relevance (García-Carmona, 2021a), and they are difficult to combat with epistemic arguments (Fackler, 2021) because they tend to have little respect for scientific evidence (Erduran, 2021).

Therefore, it continues to be an essential challenge to design, implement, and evaluate educational proposals aimed at helping basic level students to acquire an informed understanding of NOS (Akerson et al., 2011). Nonetheless, for this to become normalized in science classes, understanding basic aspects of NOS and its pedagogy should also be a primary objective in science teacher training plans (Clough et al., 2020; García-Carmona, 2021b), especially those related to preservice basic educational level teachers, whose understanding of NOS tends to leave considerable room for improvement (Capps & Crawford, 2013; García-Carmona, 2021c; García-Carmona & Acevedo 2016a).

A resource of great pedagogical interest for learning NOS is to read science news found in the media (Demirdöğen & Aydın-Günbatar, 2021; García-Carmona, 2014, 2021a; Shibley, 2003). Science-related news is relatively easy to find and provides an ideal context for thinking about and discussing certain aspects of NOS. One of the issues that received explicit media attention during the COVID-19 pandemic was how scientists do research, and whether there really is something called *the scientific method* (e.g., Cowles, 2020; Diéguez, 2020). Taking advantage of this opportunity, it was decided to analyze the effectiveness of an activity designed for preservice primary teachers (PPTs) to reflect on and learn about this aspect of NOS, using as context the reading of various news releases dedicated to it. This decision was first encouraged by some previous studies that had successfully implemented this resource in the initial training of science teachers on NOS (Cakmakci & Yalaki, 2012; García-Carmona & Acevedo, 2016b; Shibley, 2003) and second, it is because very few studies have analyzed the effectiveness of specific classroom activities that focused on improving students’ or preservice teachers’ views on the variety of methods that scientists use in their research. Moreover, no previous study was found that proposes and analyzes the pedagogical effectiveness of reading news items published in the media, specifically dedicated to the nature of methods of science, in order to reflect and learn about this aspect of NOS.

## The Myth of *the Scientific Method* in Science Education

One of the most characteristic features of NOS is the wide variety of methods that scientists use to carry out their research into the behavior of nature (Acevedo-Díaz & García-Carmona, 2017; Ioannidou & Erduran, 2021; Reif-Cox 2020). A very illustrative example of this can be found in the passage in the history of science referring to the elucidation of the molecular structure of DNA. While Rosalind Franklin approached the problem with a methodological approach oriented essentially towards experimentation, James Watson and Francis Crick did so with one based on the construction of models (García-Carmona, 2021c). Scientists are influenced by their research discipline, beliefs, attitudes, skills, creativity, and resources available, among other factors, in their choice and implementation of different methods (García-Carmona, 2021d; García-Carmona & Acevedo-Díaz, 2018). Sometimes they do make discoveries by chance or unexpectedly (serendipity), discoveries which may also be key in the development of science (Copeland, 2019). Therefore, it can be said that *the scientific method*, in the sense of an algorithmic and universal method that all scientists follow in their research, does not exist, or, in other words, is a myth (McComas, 1998; Woodcock, 2014).

It is quite a different thing that certain scientific practices and methodological rules applied in a lot of research are recognizable (García-Carmona & Acevedo-Díaz, 2018; Irzik & Nola, 2011; NSTA 2020) or common to different fields of science (Sober, 2015). The procedures or ways of approaching a research problem to a large extent depend on its nature. Indeed, some research procedures are specific or exclusive to certain sciences and unfeasible in others. For example, while in biochemical research many of the phenomena studied are usually easily reproducible in the laboratory, in other fields such as geology or astronomy, this is impossible. Therefore, the philosophy of science speaks of *family resemblance* to refer to processes or features that different sciences share (Irzik & Nola, 2011).

Research in science education has been warning for years about the negative consequences of promoting the idea among students that there is a standard method, called *the scientific method*, with which all scientific knowledge is developed (Hodson, 1996; Woodcock, 2014). In addition to promoting a distorted image of scientific activity (McComas, 1998; García-Carmona & Acevedo-Díaz, 2018), its adaptation to the educational environment has generally led to students carrying out inquiry activities mechanically and thoughtlessly, by going through an ordered series of steps (Tang et al., 2010; Windschitl et al., 2008). Despite this, the idealized and simplistic representation of scientific research being done following *the scientific method* is still quite ingrained in science teachers themselves (Ioannidou & Erduran, 2021; Vázquez-Alonso et al., 2013; Windschitl et al., 2008), in the official school science curricula (García-Carmona et al., 2014), and in the textbooks used to teach science (Binns & Bell, 2015; Reif-Cox 2020). In the context of Spain, for example, the official science curriculum for compulsory education establishes a cross-sectional block of content with one entry named: “The scientific method: its stages” (Ministry of Education 2015, p. 258). Two of the learning standards associated with this content indicate that students must “Recognize and identify the characteristics of the scientific method” and “Develop small inquiry projects in which the application of the scientific method is put into practice (…)” (p. 258). Similarly, a recent analysis of the vision of NOS given in current Spanish science textbooks for the said educational stage (Ibáñez et al. 2019) found that scientific research continues to be presented as a univocal and rigid action, organized around a series of standard stages which make up *the scientific method*.

## Nature of Methods of Science in Initial Primary Teacher Education

Various studies reveal that PPTs tend to have poorly informed views about NOS (e.g., Abell et al., 2001; Akerson et al., 2006; García-Carmona & Acevedo, 2016a; Murcia & Schibeci, 1999). However, few studies have diagnosed PPTs’ views of the nature of methods of science (e.g., Gusasola & Morentin 2007; Vázquez-Alonso et al., 2013), which also point out that the PPTs have inadequate conceptions in this regard. For example, they tend to believe that the best scientists are those who follow *the scientific method* in their research (Vázquez-Alonso et al., 2013), so they do not consider in their reflections on scientific methodology diverse and flexible strategies to solve scientific problems (Guisasola & Morentin, 2007).

Consequently, in the initial training of basic-level science teachers, it is necessary to promote reflection about *the scientific method* and its limitations that are representative of scientific activity. The purpose should be to improve the preservice teachers’ meta-scientific understanding of the practices and processes that scientists follow in their research, with a view to making suitable didactic transpositions for coherent integration into the school science curriculum (García-Carmona & Acevedo-Díaz, 2018; Ioannidou & Erduran, 2021).

Studies on the effectiveness of teaching interventions to improve the PPTs’ understanding of NOS can be found in the literature (e.g., Abell et al., 2001; Akerson et al., 2006; Bell et al., 2011). However, as in the case of the diagnostic studies mentioned above, there also are very few studies that have implemented and evaluated the effectiveness of classroom activities aimed at improving PPTs’ conceptions of the nature of methods of science. For example, García-Carmona (2021c) asked PPTs to reflectively read the historical passage on the elucidation of DNA in order for PPTs to assimilate that scientists can approach the same problem using different research methods.

Therefore, it can be said that there is a significant gap between theoretical or diagnostic studies on conceptions concerning the nature of methods of science and those aimed at improving the understanding of PPTs on this topic through ad hoc training activities. And, as mentioned at the beginning, a good resource for this can be reflective reading of science-related news items published in the media that deal with the nature of methods of science.

## Mass Media and Teaching of NOS

It is common to find news items about scientific issues and their social impact in the media. This has been especially clear during the COVID-19 pandemic (Demirdöğen & Aydın-Günbatar, 2021; García-Carmona, 2021a). Numerous news items have been published in the media and statements posted on social networks in which the work of the scientific community was the subject of analysis, criticism, and debate (López et al., 2020). This is something to take seriously into account because the information published in the media generates states of opinion in the public about the scientific or socio-scientific issue in question, and about scientific activity in general (Hodson, 2008; Jarman & McClune, 2007). For the majority of citizens, the media are their main source of information about science-related matters (The Spanish Foundation for Science & Technology 2018). Therefore, communication about science in the media should receive important attention in the processes of scientific literacy for the citizens of these times (Höttecke & Allchin, 2020; Howell & Brossard, 2021).

This educational need is echoed, for example, in the theoretical framework for the evaluation of scientific competence in the PISA tests (OECD 2019), by distinguishing a component called “Interpreting data and evidence scientifically,” which includes among its indicators “Evaluating scientific arguments and evidence from different sources (e.g., newspaper, Internet, journals)” (p. 105). The official science curriculum for the compulsory stage of education in Spain is also sensitive to this, and explicitly establishes among its educational objectives that students must “Interpret information about scientific issues of an informative nature that appear in publications and communications media” (Ministry of Education 2015, p. 258).

It is important to point out that the assimilation and evaluation of the messages about science published in the media would be favored if their readers had an informed understanding of NOS (García-Carmona, 2021a). This understanding, in accordance with the essential purposes of education about NOS (Bybee et al., 1991; Driver et al., 1996; NSTA 2020), would allow critical analysis of the content covered in the news using knowledge about how science functions and its limits, as well as its relationships with the socio-cultural context in which it is embedded. This is of substantial relevance in the analysis of socio-scientific issues (Sadler et al., 2004) where the evidence is usually interpreted in different ways, thus requiring an ability to assess its origin, scope, and reliability. To promote the development of this competence in students, reading science news in an educational context should not be limited to understanding the vocabulary and storing the message, but should also favor the identification and analysis of the features characteristic of science that are revealed (García-Carmona, 2014). This must also be done bearing in mind the intention and credibility of the authorship of the news item (Oliveras et al., 2013). For all these reasons, it is necessary that the reading be accompanied by explicit, carefully formulated, questions to invite the students to think and discuss the aspects of NOS that emerge in such news items (García-Carmona, 2021a; García-Carmona & Acevedo, 2016b).

## Research Questions

Based on all the above, the research questions that guided this study were the following:

- (1) With what conceptions about the nature of methods of science do the PPTs begin their training into the basic notions of NOS and its pedagogy?
- (2) How do the PPTs’ conceptions about the nature of methods of science progress after reading and reflectively discussing the content of various opinion pieces about the topic, published in the media?

## Methods

### Participants

The study was carried out with 52 PPTs (67% women and 33% men; aged between 19 and 28 years; mean age 19.7 years; SD = 1.7 years) who made up a group-class of the subject of science teaching (9 teaching credits) corresponding to the 2nd year of the Degree in Primary Education at the University of Seville. Given that the study had to be carried out within the academic period established for the subject and in accordance with its program, the participants selected were those to whom the researcher (and class-group educator) had access at the time of conducting this study (convenience sample). The commonest profile of the PPTs taking the subject in this degree at the University of Seville can be summarized with the following features (García-Carmona & Cruz-Guzmán, 2016c): students (1) who have had an unsatisfactory school experience with science, (2) who come to the university degree through humanities and social science academic paths (and therefore have a fairly limited background in school science), and (3) who, being preservice primary school teachers, are usually not very interested in teaching science.

The objectives of the science teaching subject are to (i) reflect on and understand the purpose of basic science education, (ii) analyze the primary education school science curriculum according to the suggestions from research in science education, (iii) know what the students’ usual conceptions and difficulties in learning science are, (iv) become familiar with resources and strategies for science education and evaluation, and (v) learn to design plans and activities to teach science in primary education. Training the PPTs in basic notions of NOS and its pedagogy is part of objective (ii). It should be added that the PPTs arrive at the subject without having received any prior instruction in it.

### Description and Implementation of the Activity

The activity carried out is part of a unit about basic notions of NOS and its pedagogy. It consisted of a reflective reading of the three articles indicated below, in which their authors (history and philosophy of science researchers) reflect upon methods of science with the use of a language accessible to the general public. Two of the news items were published during the COVID-19 pandemic, and the other a few years before, although it was considered interesting to also be included in the activity as complementary reading to the other more recent items. It should be noted that the articles were purposively selected by the educator from a multitude of science-related media news, without carrying out a systematic search. The only selection criterion used was to have several articles published in the media that explicitly dealt with *the scientific method*, so that reading them would encourage the PPTs to reflect on it. In this sense, the fact that the articles were written by philosophers and historians of science was not a requirement for the selection, but a result of that search (coincidentally, the articles found were written by these academics).

- Article 1: *Does ‘The scientific method’ exist? Philosophy and science in the twenty-first century* (Diéguez, 2020). The author is a professor of philosophy of science at the University of Málaga (Spain). He begins his reflection by responding to the question posed in the title with a resounding “no.” To argue this, he makes a brief review of different methods proposed throughout the history of science (inductive, hypothetical-deductive, and abductive), citing illustrious philosophers of science such as F. Bacon, R. Descartes, W. Whewell, P. Feyerabend, M. Bunge, and C.S. Pierce, among others. He emphasizes that “Systematicity and rigor by themselves do not make something scientific,” and that “It is not necessary to have a set of fixed and universal rules exclusive to science to form a clear idea of what science is.”
- Article 2: *The scientific method can’t save us from the coronavirus* (Cowles, 2020). The author is an assistant professor of history at the University of Michigan. He begins his reflection by stating that “The scientific method can’t save us—because it doesn’t exist.” In the context of the COVID-19 pandemic, he criticizes the continuous talk in the media and social networks about *the scientific method*, when it is well known that what scientists do, individually and collectively, is too diverse, dynamic, and therefore difficult to summarize in a “recipe.” He then argues that if science saves us, it will be precisely because it does not use a single method. In his reflection, he uses some of the ideas of the philosopher, psychologist, and pedagogue J. Dewey about how people think.
- Article 3: *There is no scientific method* (Blachowicz, 2016). The author is a professor emeritus of philosophy at the Loyola University Chicago. When he states that there is no scientific method, what he really means is that there is no method exclusive to science. He reflects on the parallelism that can be established between the composition of a poem and the resolution of a scientific problem. He concludes that the essential difference is that science deals with highly quantified variables, and that the precision of its results is what provides reliability. He also emphasizes that not only observed facts are what guide scientists’ theories. He criticizes, therefore, that in science education, there is the projection of the idea that the construction of scientific knowledge is primarily guided by its empirical fit.

Because of the time available for the activity was limited within the program of the subject, and that the PPTs were not used to dealing with NOS topics, the most viable option for it to be implemented was through a short teaching intervention (García-Carmona, 2021c; Leach et al., 2003; Williams & Rudge, 2016). The learning objectives were for the PPTs to (1) review their preconceptions about the nature of methods of science, (2) reflect on and discuss the most characteristic features of these methods in the context of news content, and (3) understand that *the scientific method* is a simplistic and limited idealization of methods of science and does not represent the wide range of methods and processes that scientists use in their research.

Before starting the activity, the PPTs responded individually to a questionnaire so as to diagnose their conceptions about characteristic features of methods of science (pre-test). Then, they were organized into small working groups to carry out the activity. This was done to favor the elaboration of fuller and more reflective responses since each group had to strive to give an answer that was the result of discussion and consensus among its members. The activity was implemented in the following three phases:

- *Initial phase.* Once the PPTs had read the news individually, in class, they responded as a group to the questions in Table 1. As indicated, the educator encouraged each group to make its responses to the questions be the result of discussion and consensus among its members. The responses were prepared in a 2-h class session. Below are examples of initial responses from two of the groups to some of the questions posed:
  Our ideas about methods of science have been altered, since we thought (…) that the scientific method was a strictly ordered set of steps and guidelines to be followed; and we have discovered that it does not have to be this way, since each scientific team, or even each scientist follows different steps, depending on the idiosyncrasy of their science, the main common element being (…) experimental and theoretical rigor. (Initial response of one of the groups to question 1 of the activity)
  The procedure carried out by science and the one carried out when composing a song are similar (…) The only difference is that in science it is based on the experience gathered from observing phenomena and in artistic expression it is based on experiences and life, in an emotional sense. (…) The results of science are more reliable, but not due to the procedure carried out, which is shared by other areas such as poetry, but because of the quantitative nature of the variables that science deals with as opposed to poetry. (Initial response of one of the groups to question 2 of the activity)

**Table 1:** Questions to reflect on the nature of methods of science based on the readings of the selected news items

1. After reading the news items, explain if your ideas about methods of science have been reaffirmed or altered, and why
2. According to what you have read, what differences do you find in the procedures followed when composing a song (lyrics and music) and in the search for answers to a research problem about a natural phenomenon?
3. In the context of what you have read in the news, what methodological differences do you think need to be taken into account when researching into “the development of a medicine” and “the determination of a species of dinosaur”?
4. What role do you think the scientists’ creativity and imagination play in the development of their research?
5. If two teams of scientists follow exactly the same method when researching a problem, do you think they would reach the same conclusions? Give arguments for your response
6. If two teams of scientists follow different methods when researching the same problem, do you think they would reach the same conclusions? Give arguments for your response

- *Intermediate phase.* The groups shared and discussed their responses in a 90-min class session. The educator moderated the discussion between the groups, made clarifications when necessary, and posed additional questions to deepen or redirect the discussion in order to enrich the exchange of opinions. When a group presented unfounded or misinformed ideas about the topic being analyzed, the educator tried to provoke some cognitive conflict so that they would reconsider their arguments. The purpose was to encourage the groups to reach common conclusions regarding the topic discussed. This was done without any indoctrination by the educator since it was important to know what levels of understanding the PPTs were capable of reaching as a learning community.
- *Final phase.* After the groups had shared and discussed their opinions regarding the questions posed, each reviewed its initial responses, and introduced all the corrections, exceptions, or extensions they considered necessary to improve their arguments. They had a week deadline outside class to do this. These responses, together with the initial ones, were recorded by the groups in a report that they submitted to the educator as part of their evaluation of the subject (the groups’ records in their reports were not analyzed in this study for reasons of length). Examples of the groups’ responses after the sharing and discussion in class are as follows (the underlining indicates what was added to the initial response):

Although they [both] share the beginning and the process, the end result is different as the scientist reaches an objective conclusion. (…) The musician reaches a more subjective result since they observe reality in a more abstract way and make it go through other filters linked to personal emotions and feelings. Scientific observation can also be subject to the interpretation given to it by each scientist and, therefore, the observation of the same phenomenon by two different scientists does not have to give an identical result… [But they] … will have to adapt to certain scientific rules… and [may be] refuted by the rest of the scientific community. (…) In the case of songs, the result obtained is an artistic manifestation subject to free interpretation. (Final response of one of the groups to question 2 of the activity; brackets added)

With respect to the methods, [in the development of a medicine, the study] can be replicated as many times as is necessary thanks to volunteers and be analyzed in the laboratory (products, substances are tested and demonstrated). Nonetheless, dinosaurs cannot be experimented on since they are extinct. Something that we can add as a difference between the two is what is at risk with each one, since the significance is very different. (…) If the result is wrong, there will not be the same consequences for the two cases. Obviously, an error when determining a species of dinosaur does not affect society in the same way as if a failure occurs in a vaccine that makes it, for example, harmful or have worrying side effects. In addition, different solutions can be found to solve problems in the case of medicine, such as vaccines. (Final response of one of the groups to question 3 of the activity)

The PPTs responded to the questionnaire again (post-test) one week after submitting the activity report.

### Instrument

To diagnose the PPTs’ initial conceptions (pre-test) and evaluate their progression after the activity (post-test), three questions (70,721, 90,611, and 90,621)^1^ were used from the *Opinions about Science, Technology and Society Questionnaire* (COCTS in its Spanish acronym) (Vázquez et al., 2006; Vázquez-Alonso et al., 2013). These refer to methods of science and will here be denominated as follows: (1) *How do scientists do research*; (2) *What is the scientific method*; and (3) *What applicability and effectiveness does the scientific method have*.

Each question in the questionnaire includes a series of statements to which the respondent must show their degree of agreement, using a Likert scale from 1 to 9. The statements are classified into three categories: adequate (A), plausible (P), and naïve (N). The metric used for the analysis of the responses is summarized in Table 2. Each direct score (from 1 to 9) is then transformed into a normalized index within the interval [− 1, + 1] using a scaling procedure that takes into account the category of the statement (adequate, plausible, naïve). Thus, for a declaration classified as adequate, total agreement with this (9) is assigned the index “ + 1,” while total disagreement (1) is assigned the index “ − 1”. Intermediate indices are calculated proportionally. For a naïve statement, the scale assigns a scoring index that is inverse to that of the adequate statements (i.e., “ + 1” to 1, and “ − 1” to 9). For a plausible statement, the scoring index assigned is + 1 to the median direct score (5) and “ − 1” to the two extremes (1 and 9); the rest are calculated proportionally.

**Table 2:** Correspondence between direct scores and scores of beliefs (A, P, N), according to the category (Adequate, Plausible, or Naïve) of each sentence, and computation of the normalized index of the sentence (A’, P’, N’) (Taken from Vázquez-Alonso et al., 2013)

Direct score scale Degree of agreement										
	Total	Near Total	High	Partial High	Partial	Partial Low	Low	Near Null	Null	
	9	8	7	6	5	4	3	2	1	
Correspondence to scores of beliefs										
Categories	Direct scores of beliefs (A, P, N)									Normalized index [-1, +1]
Adequate	4	3	2	1	0	− 1	− 2	− 3	− 4	A’=A/4
Plausible	-2	− 1	0	1	2	1	0	− 1	− 2	P’=P/2
Naïve	− 4	− 3	− 2	− 1	0	1	2	3	4	N’=N/4

This procedure is common for Likert scales that use multidirectional statements, in order to avoid revealing the “correct position” through convergent statements (Vázquez-Alonso et al., 2013). Likewise, it allows the data to be interpreted in a relatively simple way: the closer to the maximum positive value (+ 1) an index is, the more informed the opinion of the respondent regarding the statement considered, while the closer to the negative value (− 1) the index is, the more misinformed they are.

### Data Analysis

To respond to the first research question, the COCTS was used as the diagnostic instrument (pre-test). It has been rigorously validated by its proponents (Vázquez et al., 2006; Vázquez-Alonso et al., 2013). In the analysis of the data, descriptive statistics tools were applied to determine trends, strengths, and weaknesses in the opinions of the PPTs about the nature of methods of science. To determine the degree of reliability of the data, Cronbach’s α coefficient was calculated. This statistical operation suggested removing one of the seven original statements from question 1 of the questionnaire in order to ensure an appropriate degree of reliability. With its elimination, the coefficient α obtained was 0.76. Therefore, the pre-test data analyzed in this study presented an acceptably good degree of internal consistency (Ursachi et al., 2015).

With respect to the second research question, aimed at determining possible improvements in the PPTs’ understanding of the topic discussed, inferential statistical tools were applied to compare the pre-test and post-test data. Before doing so, the Cronbach’s α coefficient corresponding to the post-test data was also calculated. It was 0.71, so that these data also presented an acceptable degree of internal consistency.

To compare the pre-test and post-test data, it was necessary to use some statistical test to compare related samples. In order to select the most appropriate test, the data were first subjected to a normality analysis using the Kolmogorov–Smirnov test. With a significance level of 0.05, this test indicated that the data did not follow a normal distribution. This meant that it was not possible to apply parametric statistical tests. Thus, it was decided to apply a statistical test to compare the groups when the assumption of normality was inacceptable, in this case, the Wilcoxon signed-rank test (Fritz et al., 2012).

In order to determine whether the differences between the pre-test and post-test data were educationally relevant, the effect size (ES) was calculated. This statistical parameter complements the statistical significance and offers a more refined estimate of the scope of findings in studies with related samples such as the one presented here (Fritz et al., 2012). The ES calculations were made based on the values of the Z-test statistic resulting from the Wilcoxon test, using the formula: *ES* = *Z*/(*N*)^½^, where *N* is the total number of responses adding together the pre-test and the post-test (in this case, *N* = 104). In interpreting the ES data, the following criteria were applied (Cohen, 1988): small: ES < 0.3; medium: 0.3 ≤ ES < 0.5; and large: ES ≥ 0.5.

## Results

### PPT’s Prior Conceptions About the Nature of Methods of Science

Since the data handled were ordinal, the use of the median as a measure of central trend was chosen in the descriptive analysis of the results. In the following, the results of the analysis of the PPTs’ prior conceptions about the nature of methods of science (pre-test) are presented.

With respect to *How do scientists do research* (question 1 of the questionnaire), the PPTs overall showed ideas with negative median values (− 0.5) for half of the question’s statements (Table 3), denoting misinformed conceptions regarding the content. These misconceptions refer to believing that all scientists use *the scientific method* in their research, regardless of their context, availability of resources, socio-cultural conditions, etc., and that research methods depend primarily on the technology available. On the other hand, informed conceptions were found (positive indices) with trends located between the values 0.25 and 0.75, with respect to scientists sharing their ideas and opinions during their research, and that the way scientists do research is influenced by their academic formation, as well as by the technology available.

**Table 3:** Medians of the scores obtained in the pre-test, corresponding to question 1 ‘How do scientists do research’

(N=52)	Category	Label	Median [-1, +1]
*Q1. A team of scientists from anywhere in the world would research into the atom in basically the same way as a team of scientists from our country*			
*Scientists do their research in the same way around the world:*			
A. because science is universal. All scientists use *the scientific method* regardless of where they live	Naïve	Q1_A_(N)	− 0.50
B. because scientists share their opinions and ideas with each other	Plausible	Q1_B_(P)	0.50
*Scientists from different countries do their research differently:*			
C. because the way of doing science depends on the technology available	Plausible	Q1_C_(P)	− 0.50
D. because the way of doing science depends on the technology available, but, although scientists use different technology, they all use the same scientific method	Naïve	Q1_D_(N)	0.25
E. because the way of doing science depends on the education and technology available	Adequate	Q1_E_(A)	0.75
F. because different social conditions, resources, ideas, and culture affect everything, including the methods used by scientists	Adequate	Q1_F_(A)	− 0.50

With respect to *What is the scientific method* (question 2 of the questionnaire), it was found that the PPTs had a poorly informed understanding (negative central trend values) in six of the 10 statements included in the question (Table 4). This is reaffirmation of their belief that there is a thing in science called *the scientific method*, which represents what scientists do. Likewise, they had the inadequate conception that *the scientific method* consists of obtaining facts, theories, or hypotheses in an efficient way, through iterative processes of experimental verification until demonstrating the veracity of something. On the other hand, the PPTs stated, with a positive trend value, that *the scientific method* does not actually consist of the procedures or laboratory techniques that scientists write about in a journal. With positive but smaller trend values, the PPTs considered that this does not consist of simply taking data and controlling variables carefully, nor that the data cannot be interpreted in a different way.

**Table 4:** Medians of the scores obtained in the pre-test, corresponding to question 2 “What is the scientific method”

(N=52)	Category	Label	Median [-1, +1]
*Q2. When scientists do research, they are said to follow the scientific method. The scientific method is:*			
A. laboratory procedures or techniques; often written in a book or journal, usually by a scientist	Naïve	Q2_A_(N)	0.38
B. record data very carefully	Naïve	Q2_B_(N)	0.13
C. control experimental variables carefully, leaving no room for interpretation	Naïve	Q2_C_(N)	0.13
D. obtain facts, theories, or hypotheses efficiently	Naïve	Q2_D_(N)	− 0.50
E. check and re-check, proving that something is true or false in a valid way	Naïve	Q2_E_(N)	− 0.50
F. postulate a theory and then create an experiment to test it	Naïve	Q2_F_(N)	− 0.50
G. pose questions, make hypotheses, collect data, and draw conclusions	Plausible	Q2_G_(P)	− 0.50
H. a logical and widely accepted way of solving problems	Plausible	Q2_H_(P)	0.00
I. an attitude that guides scientists in their work	Plausible	Q2_I_(P)	− 0.25
J. a way of talking about what scientists do, but there is really no such thing as a scientific method	Adequate	Q2_J_(A)	− 0.75

Finally, regarding *What applicability and effectiveness does the scientific method have* (question 3 of the questionnaire), the trends obtained indicated that the PPTs had poorly informed understandings (negative values of the medians) in four of the five statements of the question (Table 5). The most marked naïve idea in practically all the participants (with a representative index of − 1) is the belief that chance scientific discoveries (serendipity) are very frequent. Following this, and with trends located at a somewhat smaller negative index (− 0.5), the PPTs considered that *the scientific method* ensures valid and reliable results, as well as that scientists do not use those methods that are best adapted to their research. Also, although to a lesser extent (trend with an index − 0.25), the PPTs considered that *the scientific method* taught in science classes is the one that scientists follow while doing research. The only reasonably informed idea (trend marked with a median value of 0.5) that the PPTs showed is that, although *the scientific method* may be useful or applicable in many studies, it does not ensure results.

**Table 5:** Medians of the scores obtained in the pre-test, corresponding to question 3 “What applicability and effectiveness does the scientific method have”

(N=52)	Category	Label	Median [-1, +1]
*Q3. The best scientists are those who follow the stages of the scientific method*			
A. The scientific method ensures valid, clear, logical, and accurate results. Therefore, most scientists follow the steps of the scientific method	Naïve	Q3_A_(N)	− 0.50
B. The scientific method, as taught in class, should work well for most scientists	Naïve	Q3_B_(N)	− 0.25
C. The scientific method is useful or applicable in many cases, but does not ensure results. Therefore, the best scientists also have originality and creativity	Adequate	Q3_C_(A)	0.50
D. The best scientists are those who use any method to obtain favorable results (including imagination and creativity)	Plausible	Q3_D_(P)	− 0.50
E. Many scientific discoveries were made by chance, and not following the scientific method	Plausible	Q3_E_(P)	− 1.00

### Progression of PPTs’ Conceptions About the Nature of Methods of Science

Once the PPTs had completed the activity according to the phases indicated above, they responded to the questionnaire again (post-test). The intention was to determine what learning progressions the PPTs showed regarding the nature of methods of science. The Wilcoxon signed-rank test (Table 6) showed a positive progression in 15 of the 21 statements that made up the three questions of the questionnaire. Of these progressions, 10 were also statistically significant at a significance level of 0.05. Some regression was also observed in five of the 21 statements, although of these only one case (Q1_C_ (P), which refers to methods of science depend on the technology available) was statistically significant at a significance level of 0.05.

**Table 6:** Wilcoxon signed-rank test to determine progression of PPTs’ conceptions about the nature of methods of science by comparing pre- and post-test results

Statements	Positive ranks^a^	Negative ranks^b^	Ties	Total	Z	Asymp. sig. (2-tailed)
Q1_A_(N)	40	7	5	52	− 5.019^c^	0.000*
Q1_B_(P)	18	25	9	52	− 1.194^d^	0.232
Q1_C_(P)	11	26	15	52	− 2.622^d^	0.009*
Q1_D_(N)	44	7	1	52	− 5.438^c^	0.000*
Q1_E_(A)	27	16	9	52	− 2.366^c^	0.018*
Q1_F_(A)	30	11	11	52	− 3.105^c^	0.002*
Q2_A_(N)	26	19	7	52	− 0.453^c^	0.650
Q2_B_(N)	20	27	5	52	− 0.515^d^	0.606
Q2_C_(N)	31	17	4	52	− 1.324^c^	0.186
Q2_D_(N)	27	14	11	52	− 2.367^c^	0.018*
Q2_E_(N)	28	18	6	52	− 1.451^c^	0.147
Q2_F_(N)	31	15	6	52	− 2.718^c^	0.007*
Q2_G_(P)	28	16	8	52	− 1.956^b^	0.050
Q2_H_(P)	21	21	10	52	− 0.549^d^	0.583
Q2_I_(P)	24	19	9	52	− 0.462^c^	0.644
Q2_J_(A)	47	3	2	52	− 5.506^c^	0.000*
Q3_A_(N)	43	4	5	52	− 5.465^c^	0.000*
Q3_B_(N)	32	15	5	52	− 3.197^c^	0.001*
Q3_C_(A)	26	15	11	52	− 1.565^c^	0.018*
Q3_D_(P)	17	19	16	52	− 0.410^c^	0.968
Q3_E_(P)	14	18	20	52	− 0.322^c^	0.748

^a^No. of PPTs with higher scores in the post-test than in the pre-test: positive progression.

^b^No. of PPTs with lower scores in the post-test than in the pre-test: negative progression.

^c^Based on negative ranks.

^d^Based on positive ranks.

*Significant difference between the pre- and post-test results for a significance level of 0.05.

The analysis of the PPTs’ learning progressions was complemented with calculations of the effect size (ES) (Table 7). The objective was to determine whether or not statistically significant progressions were also relevant in the context of this research study. The results of these calculations indicate that, of these progressions, six had an effect size between medium and large (ES ≥ 0.3), i.e., relevant or highly relevant educationally learning progressions. All the regressions detected, including the one that was statistically significant, presented a small or irrelevant effect size (ES ≤ 0.3).

**Table 7:** Analysis of the PPTs’ progressions in their conceptions about the nature of methods of science by calculating the effect size

(N=52)	Pre-test [-1, +1]		Post-test [-1, +1]		
Statements	Median	Rank	Median	Rank	Effect size^‡^ (ES)
Q1_A_(N)	-0.50	2	0.75	2	0.50**
Q1_B_(P)	0.50	2	0	2	− 0.12
Q1_C_(P)	0.25	2	0	2	− 0.26
Q1_D_(N)	-0.50	2	0.75	2	0.53**
Q1_E_(A)	0.25	2	0.50	2	0.23
Q1_F_(A)	0.75	2	0.75	1.75	0.30*
Q2_A_(N)	0.38	1.75	0.50	2	0.04
Q2_B_(N)	0.13	2	0	2	− 0.05
Q2_C_(N)	0.13	2	0.50	2	0.13
Q2_D_(N)	-0.50	1.25	-0.25	2	0.23
Q2_E_(N)	-0.50	1.75	-0.50	2	0.14
Q2_F_(N)	-0.50	2	0	2	0.27
Q2_G_(P)	-0.50	1.5	-0.50	2	0.20
Q2_H_(P)	0.00	2	0	2	− 0.05
Q2_I_(P)	-0.25	2	0	2	0.05
Q2_J_(A)	-0.75	2	1	2	0.54**
Q3_A_(N)	-0.50	2	0.50	2	0.54**
Q3_B_(N)	-0.25	2	0.25	2	0.31*
Q3_C_(A)	0.50	2	0.88	1.75	0.15
Q3_D_(P)	-0.50	1	-1	2	− 0.04
Q3_E_(P)	-1	1	-1	2	− 0.03

^‡^Positive effect sizes indicate greater indices in the post-test than in the pre-test, and negative effect sizes indicate the contrary.

*Medium effect size (0.3 ≤ ES < 0.5).

**Large effect size (ES ≥ 0.5).

Therefore, it can be stated that, after carrying out the activity, the PPTs showed considerable improvements in their understanding regarding the following characteristic features of methods of science:

- One cannot speak of a universal scientific method, which all scientists use in their research.
- Different social conditions, resources, ideas, and culture affect the methods scientists use in their research.
- *The scientific method* is an idealized, simplistic, and therefore poor way of representing how scientists do research (i.e., it is a myth).
- The application of *the scientific method* does not ensure valid and reliable results.
- *The scientific method* that is taught in science classes does not represent the way professional scientists actually carry out research.

## Discussion

The pedagogical efficacy of reflexively reading science news articles to learn about the nature of methods of science has been analyzed. This aspect of NOS was selected for two reasons: the first, because it took advantage of the fact that news about the subject had been published in the media due to the COVID-19 pandemic; and the second, because it is a NOS aspect about which there are deeply rooted misinformed conceptions even among science teachers, in school science curricular prescriptions, and in science textbooks (García-Carmona et al., 2014; Reif-Cox 2020; Windschitl et al., 2008). Therefore, one way to improve this educational situation is to treat this NOS content with priority during the training of PPTs.

With respect to the first research question (analysis of the PPTs’ prior ideas about methods of science), the study found that PPTs began their training in NOS with the commonest misinformed conceptions about the topic, according to the literature (McComas, 1998; Woodcock, 2014). They had assimilated the idea that there is such a thing called the scientific method, which (i) represents the way in which all scientists do their research, (ii) is independent of the contexts and circumstances in which research is carried out, except for the technology available, (iii) is applied in iterative processes until the desired result is achieved, and (iv) is the most reliable and efficient way of doing science. Nonetheless, a misconception was also detected among the PPTs which has hardly been documented at all in the literature: they thought that, although all scientists use the scientific method when they do research, many scientific discoveries are made by chance (serendipity). Therefore, the PPTs participating in the study clearly needed to improve their understanding of the nature of methods of science, above all, with a view to their teaching adequately about this aspect of NOS in the future when they are working as science teachers. It is unsurprising therefore that initiation to scientific activity constitutes a basic and transversal block of content of the science curriculum specific for primary education in Spain.

Regarding the second research question (determination of progression of PPTs’ conceptions about the nature of methods of science), after the implemented activity, the PPTs managed to progress favorably in their ideas on various characteristic features of methods of science. They ended up accepting that the scientific method is a myth (McComas, 1998; Woodcock, 2014) which does not represent the methodological diversity that scientists use in their research (Acevedo-Díaz & García-Carmona, 2017; Ioannidou & Erduran, 2021; Lederman, 2007) and that therefore its rigorous application does not guarantee success in scientific research. Consistent with this, the PPTs showed that they also understood that what is explained in science classes about the scientific method is a misrepresentation of how professional scientists actually do research (Hodson, 1996; Ibáñez et al. 2019). They also achieved a relevant progression in relation to an essential feature of the nature of methods of science: the awareness that factors such as social and cultural context, the availability of resources, and the scientists’ ideas influence the methods they choose when doing research. This feature of methods of science, largely non-epistemic in nature due to the sociological perspective it includes (García-Carmona, 2021d), helps to reinforce the prior idea that, in effect for several reasons, not all scientists address research problems in the same way.

In view of these results, it can be said that the reflective reading of certain news items about science published in the media is an effective resource to learn about NOS (Demirdöğen & Aydın-Günbatar, 2021; García-Carmona, 2014, 2021a). But this resource presents other potentialities as well as the objective of favoring the discussion and understanding of aspects of NOS. Its integration into science classes can contribute to the promotion of scientific literacy consonant with and contextualized in a hyper-informed society, where the media play a relevant role in the states of opinion that citizens have on matters related to science (Hodson, 2008; Höttecke & Allchin, 2020; Jarman & McClune, 2007).

Together with all this, it should also be noted that the activity implemented is in tune with the suggestions deriving from science education research, which indicate that the best way to learn NOS is through an explicit and reflective approach (Lederman, 2007). Indeed, the activity was designed to address NOS content with specific educational objectives which encouraged the PPTs to think about and discuss it, and whose learning results would be evaluated with an instrument (COCTS) specifically designed for this. Activities like this, which require a relatively short time to implement, have the pedagogical potential to encourage science teachers to integrate NOS content into their classes (García-Carmona & Acevedo, 2016b). This is especially important since school science programs are generally overloaded with content.

## Contributions and Limitations

The study presented here is a novel contribution to the advancement of the initial primary teacher education in the teaching/learning of NOS, which can be summarized in the following terms. First, when reviewing the literature on NOS education, it can be verified that studies that have analyzed in the classroom the pedagogical effectiveness of reading news items published in the media to learn about aspects of NOS are quite scarce, and even more if it is referred to the initial primary teacher education.

Second, the activity presented in this study, which aims to reflect and learn about the nature of methods of science using science-related news items in the media, is also innovative. Although the “myth of the scientific method” has been the subject of various analyses in the literature on NOS education (some works on it are cited in this manuscript), most of these studies focus on this issue from a theoretical approach or are limited to diagnosing the views of students and teachers on this topic. Very few studies have analyzed the effectiveness of specific classroom activities focused on improving students’ and preservice teachers’ views on the variety of methods that scientists use in their research. Furthermore, no previous studies were found that propose and analyze the pedagogical effectiveness of reading news items specifically dedicated to the nature of methods of science to reflect and learn about this aspect of NOS.

Third, unlike most studies on the implementation and evaluation of educational proposals in the science classroom for learning about NOS issues, the proposal presented in this study is characterized by being a short intervention that facilitates its integration into generally overloaded school science programs (García-Carmona, 2021c). The latter is particularly relevant to be pointed because longer teaching interventions do not seem to be more pedagogically effective in improving the understanding of NOS in the preservice teacher education (Williams & Rudge, 2016).

In addition, like all studies carried out with samples of participants chosen for convenience, the results presented here cannot be generalized to the entire population of PPTs. Nonetheless, they can serve as a stimulus so that other PPT educators are encouraged to promote the reading of science news items published in the media as a pedagogical resource in their teacher training plans about NOS, especially if their PPT training contexts have similar characteristics to those of the participants in this study.

On the other hand, the learning that the PPTs acquired about the nature of methods of science was probably not broad and robust. Among other reasons, assimilating a metaknowledge about science such as that has been addressed here with a single teaching intervention, regardless of whether it is longer or shorter, is an always difficult educational challenge, especially if the trainees start from deep-rooted misconceptions about the topic. In any case, it was not possible to follow up later to determine whether the degree of assimilation of the ideas acquired by the PPTs lasted beyond the intervention period. Consequently, what one could say is that with the activity, an acquisition of initial learning was achieved that is sufficiently conducive for the PPTs to begin to banish from their heads and, therefore, from their didactic designs, inadequate conceptions regarding the nature of methods of science. In this sense, it would be interesting in subjects of science teaching in teacher education for the understanding of basic aspects of NOS and its pedagogy to be conceived of as a cross-cutting issue. In that way, the approach will be in the context of the objectives of science education, the content of school science, the strategies, and resources for the teaching *of* and *about* science, as well as the methods and instruments to evaluate the learning *of* and *about* science.

Another aspect of this study that could be questioned is the fact that the articles selected to read and reflect upon the nature of methods of science were written by philosophers and historians of science. Although it is true that normally those who reflect on aspects of NOS are academics who belong to such disciplines, it would be good to complement these readings with other science news items (if it is possible to find them) that are also conducive to reflection on the topic, but do not provide any philosophical position on the matter. In the same way, the reading of news about contemporary science could be complemented with that of some passages from the history of science which favor reflection on this aspect of NOS (García-Carmona, 2021c). Such a combination of readings can provide the student with a broader and more authentic vision of how scientists have done their research throughout history (Matthews, 2012), with the use of different methods being one of the most characteristic features (Acevedo-Díaz & García-Carmona, 2017; Ioannidou & Erduran, 2021; Woodcock, 2014). These readings could also be combined with activities of school-level scientific inquiry, which give the students a certain freedom in planning how to approach the problem, and in which questions are raised explicitly to reflect on how different methods can lead to the same solution, or how two groups following the same method can reach different conclusions.

As future research perspectives, an attempt will be made to analyze the effectiveness of the above proposals in order to improve the training of PPTs about NOS and its pedagogy. This, however, will have to be done within the limitations currently imposed by the usual primary teacher training plans in relation to science teaching. This will doubtlessly be a great challenge for science teacher educators, but, above all, it will be important that both those responsible for designing science curricula and the authors of school science textbooks join this educational cause. Otherwise, it will be difficult to get informed conceptions about the nature of science research promoted in science classrooms. The hope is that this study can also serve as a stimulus to these two groups who are essential for science education.

## Footnotes

1 These are the labels of the three questions selected from the COCTS, but in this study, they will be denominated Q1, Q2, and Q3, respectively.
